# Unmasking Subclinical Right Ventricular Dysfunction in Type 2 Diabetes Mellitus: A Speckle-Tracking Echocardiographic Study

**DOI:** 10.3390/medicina61091516

**Published:** 2025-08-23

**Authors:** Laura-Cătălina Benchea, Larisa Anghel, Nicoleta Dubei, Răzvan-Liviu Zanfirescu, Gavril-Silviu Bîrgoan, Radu Andy Sascău, Cristian Stătescu

**Affiliations:** 1 Internal Medicine Department, “Grigore T. Popa” University of Medicine and Pharmacy, 700503 Iași, Romania; benchea.lauracatalina@yahoo.com (L.-C.B.); nicoletadubei@yahoo.com (N.D.); silviubirgoan@gmail.com (G.-S.B.); radu.sascau@umfiasi.ro (R.A.S.); cristian.statescu@umfiasi.ro (C.S.); 2 Cardiology Department, Cardiovascular Diseases Institute “Prof. Dr. George I. M. Georgescu”, 700503 Iași, Romania; zanfirescu_razvan-liviu@d.umfiasi.ro; 3 Physiology Department, “Grigore T. Popa” University of Medicine and Pharmacy, 700503 Iași, Romania

**Keywords:** type 2 diabetes mellitus, right ventricular dysfunction, speckle-tracking echocardiography, early cardiovascular risk stratification

## Abstract

*Background and Objectives*: Type 2 diabetes (T2DM) substantially increases cardiovascular risk; beyond the well-recognized left-ventricular involvement in diabetic cardiomyopathy, emerging data indicate subclinical right-ventricular (RV) dysfunction may also be present. This study aimed to evaluate whether speckle-tracking echocardiography identifies subclinical right-ventricular systolic dysfunction in type 2 diabetes, despite normal conventional indices and preserved global systolic function. *Materials and Methods*: We conducted a cross-sectional, single-center study in accordance with STROBE recommendations, enrolling 77 participants, 36 adults with T2DM, and 41 non-diabetic controls, between December 2024 and July 2025. All participants underwent comprehensive transthoracic echocardiography, including conventional parameters (tricuspid annular plane systolic excursion (TAPSE), tricuspid annular systolic velocity (TV S’), right ventricular fractional area change (RVFAC)) and deformation imaging (right ventricular global longitudinal strain (RV GLS), right ventricular free wall longitudinal strain (RVFWS)) using speckle-tracking echocardiography. Biochemical and clinical data, including glycosylated hemoglobin (HbA1c), were recorded. Correlation and ROC curve analyses were performed to explore associations and predictive value. *Results*: The mean age was comparable between the two groups (62.08 ± 9.54 years vs. 60.22 ± 13.39 years; *p* = 0.480). While conventional RV parameters did not differ significantly between groups, diabetic patients had significantly lower RV GLS (−13.86 ± 6.07% vs. −18.59 ± 2.27%, *p* < 0.001) and RVFWS (−15.64 ± 4.30% vs. −19.03 ± 3.53%, *p* < 0.001). HbA1c levels correlated positively with RV strain impairment (RVFWS r = 0.41, *p* < 0.001). Both RV GLS and RVFWS were independent predictors of RV dysfunction in logistic regression analysis. ROC analysis showed good diagnostic performance for RV GLS, AUC = 0.84 with an optimal cut-off −17.2% (sensitivity 86.1% and specificity 80.5%) and RVFWS, AUC = 0.76 with cut-off −17.6% (sensitivity 77.8; specificity 80.5%) in identifying early myocardial involvement. *Conclusions*: RV systolic dysfunction may occur early in T2DM, even when traditional echocardiographic indices remain within normal limits. Speckle-tracking echocardiography, particularly RV GLS and RVFWS, offers sensitive detection of subclinical myocardial impairment, reinforcing its value in early cardiovascular risk stratification among diabetic patients.

## 1. Introduction

Diabetes mellitus (DM) is a common chronic, multisystemic metabolic disease and represents one of the most important cardiovascular risk factors [1,2]. It is estimated that by 2040, 642 million people will suffer from DM, representing a burden for both patients and healthcare systems [3,4]. Diabetic patients are at increased risk of developing heart failure (HF). Each 1% increase in HbA1c is associated with an 8% increase in the risk of HF among patients with T2DM independent of coronary artery disease (CAD), hypertension, age, and obesity [3].

Thus, in 1972, Rubler et al. first defined diabetic cardiomyopathy (DCM) [2,3]. DCM has four stages of evolution, from an asymptomatic patient with risk factors (Stage A) to refractory HF (Stage D) [3]. DCM involves both ventricles, and the mechanisms include impaired calcium transport, accumulation of advanced glycation and products, impaired glucose metabolism, abnormal metabolism, activation of the renin–angiotensin–aldosterone system (RAAS), and inflammation [5]. Recent studies have demonstrated that energy metabolism disorders are closely linked to chronic inflammation, which plays a crucial role in cardiomyocyte apoptosis during the progression of diabetic cardiomyopathy. Imbalances in carbohydrate metabolism determine the activation of white blood cells and the recruitment of neutrophils, monocytes, and macrophages to the heart. Hyperglycemia also increases the release of proinflammatory cytokines, such as interleukin 1β (IL-1β), IL-6, IL-18, tumor necrosis factor alpha (TNF-α), and transforming growth factor β1 (TGF-β1), which favor cardiac myocyte apoptosis and the development of diabetic cardiomyopathy. Persistently elevated glucose levels lead to an increase in reactive oxygen species (ROS) and the NLPR3 inflammasome, leading to inflammation, myocardial fibrosis, cardiomyocyte apoptosis, and exacerbation of diabetic cardiomyopathy [6,7]. Most studies have analyzed the involvement of the left ventricle (LV) in DCM. But several studies have also demonstrated right heart damage due to systolic and diastolic dysfunction of the right ventricle (RV) and pulmonary pressure damage [1,8].

Zhang Y. et al. conducted a study that included 207 patients with DM and 84 patients without DM. Diabetic patients were divided into two subgroups: those with controlled diabetes (HbA1c < 7%) and those with uncontrolled diabetes (HbA1c ≥ 7%). Diabetic patients had lower values for right ventricular free wall longitudinal strain (RVFWLS) and right ventricular ejection fraction (RVEF) compared to those with normal glucose metabolism. The subgroup with uncontrolled diabetes presented lower RVFWLS values than the subgroup with controlled diabetes (*p* < 0.001). No significant differences were observed in RVEF between the two subgroups [1].

Another study demonstrated that subclinical changes in the RV can be identified by two-dimensional speckle-tracking echocardiography (2D STE). Wu T. et al. compared 52 diabetic patients with a control group of 49 healthy individuals. The right ventricular global longitudinal strain (RVGLS) and RVFWLS were lower in the diabetes group (16.85 ± 4.01% vs. 20.80 ± 1.96%, respectively, 20.79 ± 4.92% vs. 25.63 ± 4.58%, *p* < 0.001). HbA1c was an independent risk factor for the occurrence of RV subclinical changes in diabetic patients [9].

Given that early recognition of RV involvement enables timely therapy and tighter glycemic control may slow myocardial injury, we assessed RV structure and function in type 2 diabetes using conventional echocardiographic indices (TAPSE, TV S’) and speckle-tracking-derived strain to determine whether subclinical RV dysfunction is detectable despite preserved global systolic function. The study aimed to determine whether speckle-tracking echocardiography can identify subclinical right ventricular dysfunction in patients with type 2 diabetes despite preserved global systolic function.

## 2. Materials and Methods

### 2.1. Study Design

We conducted a cross-sectional, single-center study at the Cardiovascular Disease Institute “Prof. Dr. George I.M. Georgescu” in Iași, Romania, designed and reported in accordance with the STROBE (Strengthening the Reporting of Observational Studies in Epidemiology) statement (Supplementary Materials Table S1). The study aimed to assess whether early, subclinical signs of right ventricular dysfunction could be identified in patients with type 2 diabetes mellitus who maintained preserved global systolic function, compared with non-diabetic controls. This cross-sectional analysis represents the baseline evaluation of an ongoing prospective cohort, with follow-up assessments planned at 6 and 12 months. Speckle-tracking echocardiography was used to assess right ventricular dysfunction, offering a sensitive and quantitative evaluation of myocardial deformation. Data were collected between December 2024 and July 2025. No a priori sample-size estimation was undertaken due to the study’s observational nature; instead, a post hoc power analysis using G*Power 3.1.9.2 was performed to verify the ability to detect meaningful differences in right ventricular function. Study participants were identified through electronic medical records and physician referrals within the cardiology department. The study was reviewed and approved by the Ethics Committee of the “Grigore T. Popa” University of Medicine and Pharmacy in Iași (approval number 464/2024, dated 8 July 2024). All participants gave their written informed consent before entering the study.

### 2.2. Patient Selection and Data Acquisition

This study enrolled adult participants with and without T2DM to evaluate cardiac function using clinical, biochemical, and echocardiographic parameters. Data collection was conducted following predefined inclusion and exclusion criteria.

The diabetic cohort consisted of 36 adult patients (aged > 28 years) with a confirmed diagnosis of type 2 diabetes mellitus, established in accordance with the 2023 European Society of Cardiology (ESC) guidelines for cardiovascular care in patients with diabetes [10]. Diagnostic inclusion was based on at least one of the following criteria: fasting plasma glucose ≥ 7.0 mmol/L (126 mg/dL), HbA1c ≥ 48 mmol/mol (6.5%), or a 2 h plasma glucose ≥ 11.1 mmol/L (200 mg/dL) following oral glucose loading in the presence of classic hyperglycemic symptoms.

Patients were excluded if they had a history of structural or ischemic heart disease (including coronary artery disease, valvular disorders, congenital heart anomalies, cardiomyopathies), impaired left ventricular systolic function, significant arrhythmias, implanted cardiac devices, chronic pulmonary disease, pregnancy, lactation, or suboptimal echocardiographic image quality.

The control group comprised 41 non-diabetic individuals matched by age, identified through routine health evaluations. These participants had no known cardiovascular or respiratory disease. The same exclusion criteria applied to the diabetic group were enforced for controls.

Collected demographic and clinical data included age, sex, height, weight, body mass index (BMI), and body surface area (BSA), calculated using the Du Bois formula. Additional parameters included duration of diabetes (when applicable), current antidiabetic medications, smoking status, and alcohol use. Blood pressure was measured in accordance with ESC hypertension recommendations [11].

Morning fasting blood samples were drawn to evaluate key biochemical parameters: plasma glucose, HbA1c, total cholesterol, low-density lipoprotein (LDL), high-density lipoprotein (HDL), triglycerides, estimated creatinine clearance (mL/min/1.73 m^2^), and N-terminal pro-B-type natriuretic peptide (NT-proBNP).

All participants underwent a complete transthoracic echocardiographic examination using a commercially available ultrasound system. Standard 2D and Doppler echocardiographic parameters were recorded according to current American Society of Echocardiography (ASE) and European Association of Cardiovascular Imaging (EACVI) guidelines [12,13]. Right ventricular function was evaluated using both conventional and speckle-tracking echocardiographic techniques. Standard apical four-chamber views optimized for right heart assessment were acquired in all participants. Conventional parameters, including TAPSE, RVFAC, and TV S’, were measured following current ASE/EACVI guidelines [12,13]. In addition, right ventricular myocardial deformation was assessed using speckle-tracking echocardiography. All studies were analyzed offline by two experienced echocardiographers blinded to group assignments. Strain measurements were processed on a single platform (GE EchoPAC PC version 204; GE Vingmed Ultrasound, Horten, Norway), improving internal consistency but potentially limiting cross-vendor reproducibility. RV GLS and RVFWS were calculated, and all parameters were used for comparative and regression analyses to identify functional alterations related to diabetes status.

### 2.3. Statistical Analysis

Statistical analyses were performed using IBM SPSS Statistics version 23.0 (IBM Corp., Armonk, NY, USA). Descriptive statistics were used to summarize the study population. Continuous variables were expressed as mean ± standard deviation (SD), while categorical variables were presented as counts and percentages. The distribution of continuous variables was evaluated using the Shapiro–Wilk test, and when appropriate, the Kolmogorov–Smirnov test, in order to determine whether parametric or non-parametric tests should be applied. Group comparisons between patients with T2DM and non-diabetic controls were conducted using Student’s *t*-test for normally distributed variables, or the Mann–Whitney U test for non-parametric data. Categorical variables were analyzed using the Chi-square test or Fisher’s exact test, as appropriate.

To evaluate the relationship between glycemic control and right ventricular function, correlation analyses were conducted using Pearson’s or Spearman’s correlation coefficients, depending on data distribution. Univariate and multivariate logistic regression models were used to identify echocardiographic predictors of right ventricular dysfunction in relation to diabetes status (1 = T2DM; 0 = control). Multivariate models were adjusted for clinically relevant covariates, including age, sex, BMI, hypertension, renal function (creatinine clearance), and LDL-cholesterol, to account for potential confounding.

Receiver Operating Characteristic (ROC) curve analysis was applied to assess the discriminatory performance of right ventricular strain parameters—particularly RV GLS and RVFWS—in distinguishing diabetic patients at risk for early cardiac involvement. The area under the curve (AUC) was calculated to evaluate diagnostic accuracy, and optimal cut-off values were identified when applicable. A two-sided *p*-value < 0.05 was considered statistically significant throughout the analysis.

## 3. Results

### 3.1. Baseline Clinical and Echocardiographic Parameters

At enrollment, age did not differ between groups (mean age: 62.08 ± 9.54 years vs. 60.22 ± 13.39 years, *p* = 0.480). A range of cardiovascular risk factors, both traditional and lifestyle-related, were observed across the study population. The prevalence of hypertension (80.6% vs. 78.0%; *p* = 0.780), current smoking (30.6% vs. 22.0%; *p* = 0.549), and alcohol use (27.8% vs. 24.4%; *p* = 0.938) was comparable between groups. In contrast, LDL-cholesterol levels were lower in the diabetic group (110.0 ± 37.9 vs. 130.0 ± 36.0 mg/dL; *p* = 0.020). The proportion of patients with LDL-C ≥ 100 mg/dL (50.0% vs. 75.6%; *p* = 0.036) and ≥130 mg/dL (33.3% vs. 58.5%; *p* = 0.047) was also lower among diabetic patients. Overall, the baseline cardiometabolic burden was similar, minimizing confounding for echocardiographic comparisons.

BMI (30.64 ± 5.33 vs. 29.00 ± 3.41 kg/m^2^; *p* = 0.120) and BSA were similar between groups, reflecting a comparable anthropometric profile at baseline. Regarding laboratory findings, lipid profiles and NT-proBNP levels showed a broadly similar distribution between groups; however, a trend toward higher NT-proBNP values in the diabetic cohort, but the difference was not statistically significant (158.51 ± 210.50 vs. 94.86 ± 50.19 pg/L; *p* = 0.550). Additionally, creatinine clearance appeared to be lower in diabetic patients, but also without statistically significant differences between groups (83.00 ± 15.69 vs. 88.46 ± 18.78 mL/min/1.73 m^2^, *p* = 0.452).

Conventional echocardiographic indices of RV function (TAPSE, RVFAC, TV S’) were similar between groups. By contrast, speckle-tracking analysis showed lower RV GLS (−13.86 ± 6.07% vs. −18.59 ± 2.27%; *p* < 0.001) and RVFWS (−15.64 ± 4.30% vs. −19.03 ± 3.53%; *p* < 0.001) in diabetic patients (Table 1).

Measures of RV chamber size—including basal, mid, and longitudinal dimensions—as well as end-diastolic and end-systolic areas (RV EDA and ESA), showed no major differences between the groups. Similarly, time intervals related to diastolic function, such as tricuspid isovolumic relaxation time (TRIV), contraction time (TCIV), and ejection time (ET), were comparable.

NT-proBNP correlated with RV GLS in T2DM (r = 0.55, *p* < 0.001), but not in controls (r = 0.09, *p* = 0.59)—indicating higher NT-proBNP aligns with less-negative (worse) GLS. No association was observed with RVFWS (T2DM r = 0.02, *p* = 0.91; controls r = −0.01, *p* = 0.94).

### 3.2. Association Between HbA1c Levels and Right Ventricular Performance

Pearson correlation analysis showed that HbA1c correlated with RV GLS (r = 0.48, *p* = 0.006) and RVFWS (r = 0.29, *p* = 0.001). No significant correlations were observed between HbA1c and conventional parameters (RVFAC r = 0.15, *p* = 0.151; TAPSE r = 0.09, *p* = 0.116; TV S’ r = 0.27, *p* = 0.084). Given that RV strain values are negative, higher (less negative) strain values reflect worse deformation (Table 2).

### 3.3. Predictors of Right Ventricular Dysfunction Concerning Glycemic Control

Univariate logistic regression identified RV GLS and RVFWS as significant predictors of RV dysfunction in relation to glycemic control. In multivariate analysis adjusted for age, sex, BMI, hypertension, renal function (creatinine clearance), and LDL-cholesterol, RV GLS remained an independent predictor (OR 1.362; 95% CI 1.054–1.580; *p* = 0.001), as did RVFWS (OR 1.247; 95% CI 1.015–1.309; *p* = 0.015). RVFAC did not reach significance (OR 1.173; 95% CI 0.968–1.415; *p* = 0.086) (Table 3).

### 3.4. Diagnostic Performance of RV GLS and RVFWS in Identifying Early Myocardial Involvement Associated with Diabetes

**Figure 1:**
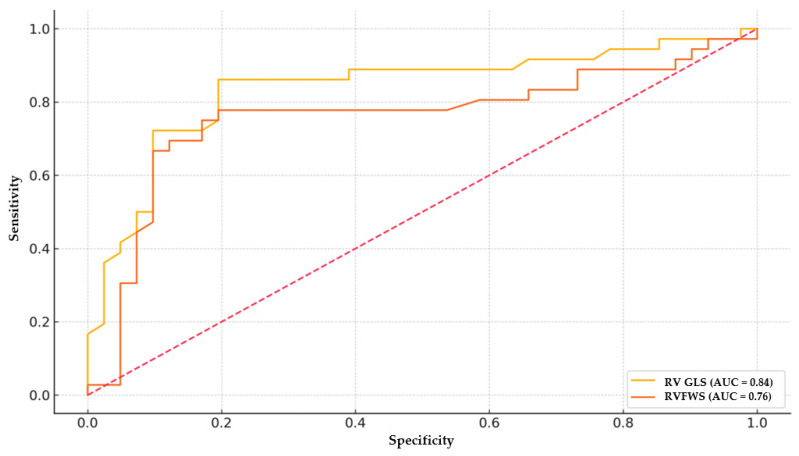
ROC curve for RV GLS and RVFWS in predicting early myocardial involvement associated with diabetes. RV GLS shows superior discrimination (AUC ≈ 0.84) compared with RVFWS (AUC ≈ 0.76). Optimal cut-offs (annotated) indicate sensitivity/specificity trade-offs near −17% for both parameters.

Receiver-operating characteristic analysis demonstrated that RV GLS had an AUC of 0.84 with an optimal cut-off value of −17.2% (sensitivity 86.1%, specificity 80.5%). RVFWS had an AUC of 0.76, with a cut-off value of −17.6% (sensitivity 77.8%, specificity 80.5%) (Figure 1).

**Figure 2:**
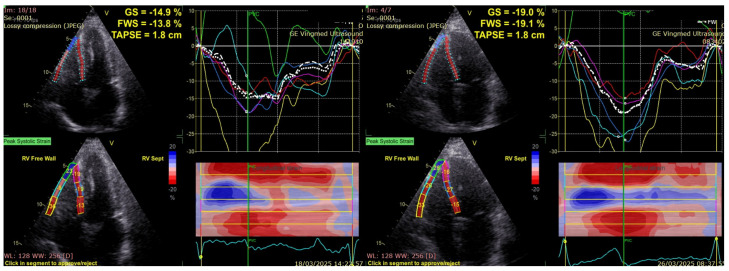
Different values of RVGLS and RVFWS for two patients with the same TAPSE value: diabetic (**left**), control group (**right**). RVGLS, Right Ventricular Global Longitudinal Strain; RVFWS, Right Ventricular Free Wall Strain; TAPSE, Tricuspid Annular Plane Systolic Excursion.

To further emphasize the limitations of conventional right ventricular function parameters, Figure 2 illustrates two representative cases, one from the diabetic group and one from the control group, which had identical TAPSE values. Despite similar conventional RV measurements, the diabetic patient showed markedly lower RV GLS and RVFWS values compared with the control, indicating impaired deformation that was not apparent on the TAPSE assessment.

## 4. Discussion

This study shows that patients with type 2 diabetes mellitus present with subclinical right ventricular systolic impairment, even when conventional echocardiographic parameters such as TAPSE, RVFAC, and TV S’ remain within normal limits. Both RV GLS and RVFWS were significantly reduced in the diabetic group, and their association with glycemic control underscores early right-sided myocardial involvement in diabetic cardiomyopathy—an aspect less frequently characterized than left ventricular dysfunction. Although research in this area is still limited, previous studies have also documented early RV alterations in diabetes, supporting the usefulness of myocardial deformation imaging [1,9]. The most pronounced abnormalities were observed in RV GLS and RVFWS, suggesting that diabetes may impair RV systolic function in the absence of structural heart disease or overt pulmonary pathology. Two-dimensional speckle-tracking echocardiography therefore represents a sensitive tool for detecting subtle functional changes that are not captured by conventional echocardiography. Early recognition of diabetic cardiomyopathy has important implications, including more accurate cardiovascular risk stratification, initiation of targeted therapies, and the possibility of delaying progression toward symptomatic heart failure.

Previous studies have demonstrated that diabetes mellitus is associated with biventricular dysfunction, with reduced right ventricular ejection fraction <30% in 15.4% of patients and LVEF < 30% in up to 40.7% of individuals with type 2 diabetes [14,15]. We observed that both RVGLS and RVFWS were significantly lower in T2DM patients, confirming subclinical dysfunction detectable only with strain imaging.

Glycemic control appears to be a stronger determinant of myocardial dysfunction than diabetes itself [16]. Zhang Y. et al. demonstrated a significantly higher prevalence of RV dysfunction in patients with poorly controlled T2DM (HbA1c ≥ 7%) compared with those with adequate glycemic control (43.1% vs. 17.6%, *p* < 0.001), indicating a direct relationship between hyperglycemia and RV performance [1]. Our study builds upon and extends these limited prior findings by providing a focused echocardiographic strain analysis of right ventricular function in diabetic patients. We observed that HbA1c correlated with both RV GLS (r = 0.48, *p* = 0.006) and RVFWS (r = 0.29, *p* = 0.001), whereas no significant associations were found with TAPSE, RVFAC, and TV S’. Because strain values are expressed as negative numbers, less-negative (higher) values indicate impaired deformation; thus, higher HbA1c levels aligned with worse RV systolic performance. This pattern underscores the greater sensitivity of deformation imaging over conventional indices for detecting early diabetes-related myocardial involvement. We demonstrated a significant correlation between HbA1c levels and both right ventricular global longitudinal strain and right ventricular free wall strain. Importantly, both parameters emerged as independent predictors of early RV dysfunction in multivariate analysis adjusted for clinical covariates. Unlike conventional parameters such as TAPSE or RVFAC, which showed no significant differences, strain imaging identified subclinical changes in RV function even in patients without overt structural abnormalities or clinical heart failure. These results underscore the novel role of RV strain analysis as a sensitive and non-invasive tool for early cardiovascular risk stratification in patients with T2DM and suboptimal glycemic control—an area that remains underexplored in the current literature.

Emerging therapeutic data also support the relevance of RV strain analysis. Clinical studies of SGLT2 inhibitors (dapagliflozin, empagliflozin) have reported improvements in RV performance, reflected in parameters such as TAPSE, TV S’, RVFAC, and RVFWS within a few months of therapy [17,18]. In our cohort, conventional indices (TAPSE, RVFAC) were preserved, yet strain analysis revealed clear RV systolic impairment in T2DM, with RV GLS and RVFWS. Together, these data suggest that RV strain, particularly RV GLS, offers a sensitive target for monitoring the myocardial benefits of SGLT2 inhibition, potentially detecting treatment response even when TAPSE and RVFAC remain normal. Medication exposure was not systematically captured in this baseline analysis. In the prospective follow-up phase of our study (planned at 6 and 12 months), we will document SGLT2 inhibitor use and test whether initiation or intensification is associated with improvements in RV strain (especially RV GLS) and better clinical outcomes.

Additional insights arise from biomarker correlations. Gastouniotis et al. demonstrated that in patients with T2DM without cardiovascular disease, higher NT-proBNP levels were associated with impaired ventricular function in T2DM patients without cardiovascular disease [19]. In our study, NT-proBNP correlated with RV GLS but not RVFWS, suggesting that strain analysis may detect subtle myocardial alterations not reflected by natriuretic peptide levels alone.

Diabetic cardiomyopathy is a distinct clinical entity that affects both ventricles and occurs independently of other common cardiovascular risk factors such as coronary artery disease, hypertension, or dyslipidemia. Its pathogenesis is multifactorial, involving altered myocardial energetics, impaired calcium handling, accumulation of advanced glycation end-products, collagen turnover abnormalities, RAAS activation, and disturbances in glucose and lipid metabolism [5,16]. While most prior studies have focused on left ventricular involvement, our study highlights a novel and underrecognized aspect—the presence of early right ventricular dysfunction detectable by two-dimensional speckle-tracking echocardiography. By identifying reductions in RVGLS and RVFWS, this study demonstrates the potential of strain imaging to serve as a sensitive, non-invasive tool for detecting subclinical RV involvement in diabetic patients. Diagnostic performance was better for RV GLS than RVFWS (AUC 0.84 vs. 0.76; *n* = 77). Clinically, RV GLS could be incorporated into routine echocardiography to flag subclinical RV systolic dysfunction in T2DM, even when TAPSE and RVFAC are normal, thereby supporting earlier risk stratification and potential therapy intensification. These insights may facilitate earlier risk stratification and therapeutic intervention, potentially slowing or preventing progression toward overt or refractory heart failure.

## 5. Study Limitations

This study has some limitations. First, the relatively small sample size limits the generalizability of findings and the statistical power of subgroup analyses. Second, the cross-sectional design precludes establishing causal relationships between glycemic control and RV dysfunction over time. Third, this was a single-center study, strain measurements were obtained on a single software platform (GE EchoPAC); while this enhances internal consistency, it may constrain cross-vendor reproducibility, and we did not formally quantify inter-/intra-observer variability. Lastly, longitudinal clinical outcomes such as hospitalization or the development of overt heart failure were not included in this initial analysis. The ongoing prospective arm with 6- and 12-month follow-up will address progression, reproducibility, and prognostic relevance of early RV strain abnormalities.

## 6. Conclusions

Our findings suggest that in patients with type 2 diabetes mellitus, right-ventricular systolic dysfunction is already present at a subclinical stage, despite normal conventional indices and absence of structural abnormalities. Speckle-tracking echocardiography identified these early changes, both RV GLS and RV FWS were reduced, with RV GLS providing the strongest diagnostic performance (AUC 0.84) and a practical threshold around −17% (sensitivity ~86%, specificity ~81%). Higher HbA1c aligned with worse RV strain, supporting a metabolic contribution to RV impairment. Integrating RV strain, particularly RV GLS, into routine echocardiographic evaluation could enhance early detection, support timely intervention, and ultimately improve outcomes in this vulnerable population.

## Figures and Tables

**Table 1:** Echocardiographic characteristics at baseline.

Variable	Patients with DM (*n* = 36 Patients)	Patients Without DM (*n* = 41 Patients)	*p*-Value
Age, mean ± SD	62.08 ± 9.54	60.22 ± 13.39	0.480
LVEF (%)	53.04 ± 5.72	54.86 ± 4.71	0.514
GLS (%)	−15.62 ± 3.15	−18.18 ± 3.66	0.136
TRIV (msec)	66.30 ± 8.65	71.97 ± 6.11	0.237
TCIV (msec)	72.16 ± 9.21	66.69 ± 7.14	0.331
ET (msec)	287.88 ± 19.51	307.77 ± 18.76	0.220
TEI index	0.43 ± 0.11	0.45 ± 0.12	0.741
E (cm/s)	71.68 ± 6.81	70.45 ± 4.86	0.831
A (cm/s)	79.40 ± 5.42	66.50 ± 4.92	0.018
E/A	0.89 ± 0.34	1.01 ± 0.29	0.204
TDE (msec)	209.36 ± 11.05	192.45 ± 10.11	0.310
MAPSE (mm)	12.63 ± 2.75	13.41 ± 2.67	0.578
TAPSE (mm)	22.72 ± 2.99	23.09 ± 2.98	0.725
RV base (mm)	34.64 ± 2.31	35.86 ± 2.10	0.397
RV mid (mm)	29.20 ± 1.22	29.40 ± 2.01	0.884
RV lenght (mm)	67.28 ± 2.13	62.04 ± 1.92	0.166
RV EDA (cm^2^)	19.64 ± 1.22	18.93 ± 1.02	0.612
RV ESA (cm^2^)	11.68 ± 0.63	11.03 ± 0.72	0.500
RV FAC (%)	42.36 ± 2.12	44.04 ± 2.41	0.168
TV S’ (cm/s)	13.04 ± 3.14	13.32 ± 1.59	0.698
RVFWS (%)	−15.64 ± 4.30	−19.03 ± 3.53	<0.001
RV GLS(%)	−13.86 ± 6.07	−18.59 ± 2.27	<0.001

A, filling due to atrial contraction; E, early diastolic filling of the left ventricle; ET, ejection time; GLS, Global Longitudinal Strain; LVEF, Left Ventricular Ejection Fraction; MAPSE, Mitral Annular Plane Systolic Excursion; RV, right ventricle; RV EDA, Right Ventricular End-Diastolic Area; RV ESA, Right Ventricular End-Systolic Area; RV FAC, right ventricular fractional area change; RVFWS, Right Ventricular Free Wall Strain; TAPSE, Tricuspid Annular Plane Systolic Excursion; TCIV, isovolumic contraction time; TDE, mitral E-wave deceleration time; TEI index, myocardial performance index; TRIV, isovolumic relaxation time; TV S’, tricuspid annular systolic velocity.

**Table 2:** Correlations between HbA1c values and right ventricular structural and functional parameters.

Variable	Pearson r Correlation Coefficient	*p* Value
RV GLS	0.48	0.006
RVFWS	0.29	0.001
RV FAC	0.15	0.151
TV S’	0.27	0.084
TAPSE	0.09	0.116

RV GLS, Global Longitudinal Strain Right Ventricle; RV FAC, right ventricular fractional area change; RVFWS, Right Ventricular Free Wall Strain; TAPSE, Tricuspid Annular Plane Systolic Excursion; TV S’, tricuspid annular systolic velocity.

**Table 3:** Logistic regression analysis to estimate predictors of right ventricular dysfunction according to diabetes control.

	Odds Ratio (95% CI)	*p*
RV GLS (%)	1.362 (1.054–1.580)	0.001
RVFWS (%)	1.247 (1.015–1.309)	0.015
RV FAC (%)	1.173 (0.968–1.415)	0.086

CI, confidence interval; RV GLS, Global Longitudinal Strain Right Ventricle; RV FAC, right ventricular fractional area change; RVFWS, Right Ventricular Free Wall Strain.

## Data Availability

De-identified study data are available on reasonable request from the corresponding author (larisa.anghel@umfiasi.ro). A justification for its further use should be provided.

## Supplementary Materials

The following supporting information can be downloaded at: https://www.mdpi.com/article/10.3390/medicina61091516/s1, Table S1: STROBE Statement—adapted checklist of items to be included in reports of observational studies.

## Abbreviations

The following abbreviations are used in this manuscript:

T2DM	Type 2 Diabetes mellitus
RV	Right ventricular
TAPSE	Tricuspid annular plane systolic excursion
TV S’	Tricuspid annular systolic velocity
RFAC	Right ventricular fractional area change
RVGLS	Right ventricular global longitudinal strain
RVFWS	Right ventricular free wall longitudinal strain
HbA1c	Glycosylated hemoglobin
ROC	Receiver operating characteristic
AUC	Area under the curve
DM	Diabetes mellitus
HF	Heart Failure
CAD	Coronary artery disease
DCM	Diabetic cardiomyopathy
RAAS	Renin-angiotensin-aldosteron system
IL	Interleukin
TNF-α	Tumor necrosis factor alpha
TGF-β1	Transforming growth factor beta 1
ROS	Reactive oxygen species
LV	Left ventricle
RVFWLS	Right ventricular free wall longitudinal strain
RVEF	Right ventricular ejection fraction
2D STE	Two-dimensional speckle-tracking echocardiography
ESC	European Society of Cardiology
BMI	Body mass index
BSA	Body surface area
LDL	Low-density lipoprotein
HDL	High-density lipoprotein
NT-proBNP	N-terminal proB-type natriuretic peptide
ASE	American Society of Echocardiography
EACVI	European Association of Cardiovascular Imaging
SD	Standard deviation
RVEDA	Right ventricular end-diastolic area
RVESA	Right ventricular end-systolic area
TRIV	Isovolumic relaxation time
TCIV	Isovolumic contraction time
ET	Ejection time
A	Filling due to atrial contraction
E	Early diastolic filling of the left ventricle
GLS	Global longitudinal strain
LVEDV	Left ventricular end-diastolic volume
LVESV	Left ventricular end-systolic volume
LVEF	Left ventricular ejection fraction
MAPSE	Mitral annular plane systolic excursion
TDE	Mitral E-wave deceleration time
TEI index	Myocardial performance index
