# Supplementary material for: Unmasking Subclinical Right Ventricular Dysfunction in Type 2 Diabetes Mellitus: A Speckle-Tracking Echocardiographic Study
Source: Medicina (Kaunas). 2025 Aug 23;61(9):1516. doi: 10.3390/medicina61091516 (PMC12471923; doi:10.3390/medicina61091516)
Supplement: Supplementary file 1 [file medicina-61-01516-s001.zip › medicina-3809731-supplementary.pdf]

STROBE Statement

Manuscript: **"Unmasking Subclinical Right Ventricular Dysfunction in Type 2 Diabetes Mellitus: A Speckle-Tracking Echocardiographic Study"**

This checklist has been completed for the above manuscript and is provided as supplementary material.

Section	Item No.	Recommendation	Location in manuscript
Title and abstract	1(a)	Indicate the study design with a commonly used term in the title or the abstract.	Title; Abstract (Background/Methods) (page 1)
Title and abstract	1(b)	Provide in the abstract an informative and balanced summary of what was done and what was found.	Abstract (Objectives/Methods/Results/Conclusions) (page 1)
Introduction	2	Explain the scientific background and rationale for the investigation being reported.	Introduction (pages 1-3)
Introduction	3	State specific objectives, including any prespecified hypotheses.	Abstract (Objectives) (page 1); Introduction (final paragraph) (page 3)
Methods	4	Present key elements of study design early in the paper.	Methods 2.1 Study Design (observational, cross-sectional) (page 3)
Methods	5	Describe the setting, locations, and relevant dates, including	Methods 2.1 Study Design (single-center), 2.2 Study population (December 2024–July 2025) (page 3)

		periods of recruitment and data collection.	
Methods	6(a)	Give the eligibility criteria, and the sources and methods of selection of participants.	Methods 2.2 Study population (inclusion/exclusion; CCTA to exclude CAD) (pages 3-4)
Methods	7	Clearly define all outcomes, exposures, predictors, potential confounders, and effect modifiers. Give diagnostic criteria, if applicable.	Methods<br>2.3 Clinical Data Collection (page 3);<br>2.2 T2DM diagnostic criteria (page 3);<br>2.3 Echocardiographic parameters (page 4).
Methods	8	For each variable of interest, give sources of data and details of methods of assessment (measurement).	Methods 2.3 Clinical Data Collection (biochemistry); Echocardiography (ASE/EACVI); LA strain (vendor/software)<br>(pages 3-4)
Methods	9	Describe any efforts to address potential sources of bias.	Methods 2.3 (blinded offline analysis by two echocardiographers); Exclusion of overt CVD; Adjustment in multivariable models (pages 3-4)
Methods	10	Explain how the study size was arrived at.	Methods 2.4 Statistical analysis (post hoc power analysis; observational design)<br>(page 4)
Methods	11	Explain how quantitative variables were handled in the analyses. If	Methods 2.4 Statistical analysis (parametric/nonparametric tests; ROC; regression; HbA1c subgroup)<br>(page 4)

		applicable, describe which groupings were chosen and why.	
Methods	12(a)	Describe all statistical methods, including those used to control for confounding.	Methods 2.4 Statistical analysis (multivariable models; p-value thresholds)<br><br>(page 4)
Methods	12(b)	Describe any methods used to examine subgroups and interactions.	Methods 3.3 (post hoc HbA1c subgroup) (pages 4-5);<br><br>2.4 (specified analyses) (pages 4-5)
Methods	12(c)	Explain how missing data were addressed.	Not applicable/No missing data reported; all enrolled participants analyzed.
Methods	12(d)	If applicable, describe analytical methods taking account of sampling strategy.	Not applicable (consecutive single-center sample).
Methods	12(e)	Describe any sensitivity analyses.	Not performed (exploratory baseline analysis).
Results	13(a)	Report numbers of individuals at each stage of study.	Results 3.1 (n=77: 36 T2DM, 41 controls)<br><br>(pages 4-5)
Results	13(b)	Give reasons for non-participation at each stage.	Not applicable (consecutive sample; exclusions predefined).
Results	13(c)	Consider use of a flow diagram.	Not included; sample description provided in text.

Results	14(a)	Give characteristics of study participants (demographic, clinical, social) and information on exposures and potential confounders.	Results 3.1; Table 1 (baseline characteristics, echocardiographic characteristics)<br><br>(pages 5-6)
Results	14(b)	Indicate number of participants with missing data for each variable of interest.	Not reported; variables complete for analyzed cohort.
Results	14(c)	Summarize follow-up time (if applicable).	Not applicable (cross-sectional).
Results	15	Report numbers of outcome events or summary measures.	Results 3.2 (strain values) (page 6);<br><br>3.3 (ORs, AUCs); Figures 1, 2; Tables 1–3<br><br>(pages 5-8)
Results	16(a)	Give unadjusted estimates and, if applicable, confounder-adjusted estimates and their precision. Make clear which confounders were adjusted for and why they were included.	Results 3.3 (adjusted effects for age, HTN, dyslipidemia; ORs/β; 95% CI)<br><br>(page 6)
Results	16(b)	Report category boundaries when continuous variables were categorized.	Results 3.3 (HbA1c categories <7% vs ≥7%) (pages 6-7)

Results	16(c)	If relevant, consider translating estimates of relative risk into absolute risk.	Not applicable (diagnostic/associative metrics reported).
Results	17	Report other analyses done—e.g., analyses of subgroups and interactions, and sensitivity analyses.	Results 3.4 (HbA1c subgroup; correlations); ROC analyses<br>(pages 7-8)
Discussion	18	Summarize key results with reference to study objectives.	Discussion (opening paragraphs)<br>(pages 8-10)
Discussion	19	Discuss limitations of the study, taking into account sources of potential bias or imprecision. Discuss both direction and magnitude of any potential bias.	Section 5. Limitations<br>(page 10)
Discussion	20	Give a cautious overall interpretation of results considering objectives, limitations, multiplicity of analyses, results from similar studies, and other relevant evidence.	Discussion (comparisons with prior work; interpretation)<br>(pages 8-10)

Discussion	21	Discuss the generalizability (external validity) of the study results.	Discussion (single-center; vendor-related reproducibility)<br><br>(page 8-10)
Other information	22	Give the source of funding and the role of the funders for the present study and, if applicable, for the original study on which the present article is based.	Back matter: Funding (none)<br><br>(page 11)
Other information	—	Ethics approval and consent to participate.	Back matter: IRB approval (464/2024), consent obtained<br><br>(page 11)
Other information	—	Availability of data and materials / conflicts of interest.	Back matter: Conflicts of Interest (none declared)<br><br>(page 11)

Notes: This checklist follows the STROBE 2007 Statement (cross-sectional studies). Where items are marked 'Not applicable', the study design did not require that element or it was not performed by design (exploratory baseline analysis).
